# A pocket-based 3D molecule generative model fueled by experimental electron density

**DOI:** 10.1038/s41598-022-19363-6

**Published:** 2022-09-06

**Authors:** Lvwei Wang, Rong Bai, Xiaoxuan Shi, Wei Zhang, Yinuo Cui, Xiaoman Wang, Cheng Wang, Haoyu Chang, Yingsheng Zhang, Jielong Zhou, Wei Peng, Wenbiao Zhou, Bo Huang

**Affiliations:** 1 Beijing StoneWise Technology Co Ltd., Haidian District, Haidian Street #15, Beijing, 100080 China; 2 Innovation Center for Pathogen Research, Guangzhou Laboratory, Guangzhou, 510320 China

**Keywords:** Computational biology and bioinformatics, Drug discovery, Structural biology

## Abstract

We report for the first time the use of experimental electron density (ED) as training data for the generation of drug-like three-dimensional molecules based on the structure of a target protein pocket. Similar to a structural biologist building molecules based on their ED, our model functions with two main components: a generative adversarial network (GAN) to generate the ligand ED in the input pocket and an ED interpretation module for molecule generation. The model was tested on three targets: a kinase (hematopoietic progenitor kinase 1), protease (SARS‐CoV‐2 main protease), and nuclear receptor (vitamin D receptor), and evaluated with a reference dataset composed of over 8000 compounds that have their activities reported in the literature. The evaluation considered the chemical validity, chemical space distribution-based diversity, and similarity with reference active compounds concerning the molecular structure and pocket-binding mode. Our model can generate molecules with similar structures to classical active compounds and novel compounds sharing similar binding modes with active compounds, making it a promising tool for library generation supporting high-throughput virtual screening. The ligand ED generated can also be used to support fragment-based drug design. Our model is available as an online service to academic users via https://edmg.stonewise.cn/#/create.

## Introduction

Molecular generative models using the three-dimensional (3D) information of target pockets have garnered increasing attention in the field of de novo drug design^1–3^. The process of drug design is generally perceived as an inherently multi-constrained optimization process. The major constraints include complementarity between the ligand and protein, regarding multiple aspects, such as shape and non-covalent interactions (NCI), and requirements of the ligand itself, including synthesizability and low strain energy binding conformation. Therefore, efficiently maintaining these constraints during the training of the molecular generative model is subject to intensive discussions. Some representative attempts include the following: (1) using autoregressive algorithms or introducing conditional tokens in the training process for models generating molecules in a sequential manner^4^; (2) leveraging generative adversarial networks (GAN) and reinforcement learning to reflect the desired bias in the output distribution^5,6^; (3) employing Bayesian optimization for the search of appropriate regions in the latent space from trained models such as variational autoencoder (VAE)^7^. These approaches all possess delicately designed architecture and have accomplished great achievements by working in an end-to-end manner in the fields of machine translation, games, and image processing. However, when applied to molecule generation for drug design, additional challenges arise, including an increased number of constraints and the lack of data. As a result, the application of end-to-end design that intends to satisfy all the constraints at one stroke by using massive amounts of training data is not as effective as in other fields. Therefore, researchers pursue solutions in two directions: (1) reducing the constraints by generating non-3D molecules, in which the molecules are represented as strings (i.e. SMILES) or graphs^8–10^; (2) expanding the dataset, for example, by generating ligand–protein complex data using docking approaches^11^. Although these attempts are inspiring, a groundbreaking strategy that can pour additional experimental data in the models is still absent. The current data expansion approaches are based on calculations heavily relying on computer-aided drug design (CADD) theory. This promotes artificial intelligence (AI) models learning from well-established rules instead of “real world” information.

There are over 120,000 high quality experimental electron density (ED) maps accumulated globally in the Protein Data Bank (PDB)^12^ over the past 60 years. However, only part of the information in these experimental EDs is used for the determination of atom coordinates, whereas other information reflecting NCI^13^, time-averaged conformational change^14,15^, and solvent distribution^16^ remains untapped. In addition to being considered as data source, ED is also an ideal representation for molecules because it naturally reflects the physical and chemical properties of a molecule. Specifically, the ED intensity isosurface describes the shape of a molecule; electron localization function (ELF)^17^ describes the bond properties; and ED topology, such as saddle points, indicates the NCI between molecules^13^. Therefore, compared to the traditional 3D molecule representations including the node-edge based representation^18,19^ and dot-cloud-based representation such as Gaussian filtering^20^ and van der Waals radius^21^, there is no need for ED to involve multiple channels to enhance the representation of physical and chemical properties, thereby avoiding the data sparsity problem. Furthermore, ED is continuously smoothing and compatible with convolutional neural networks (CNN).

Based on the forementioned reasons, we used ED as molecule representation and introduced experimental ED as training data in this work. Our model was trained to first generate ED from the pocket and then interpret the generated ED into molecules. We evaluated our model using classical CADD indicators, such as the quantitative estimate of drug-likeness (QED)^22^ and synthetic accessibility score (SAS)^23^, to test the molecular validity. Additionally, we employed a set of more intuitive indicators to test whether our model could generate novel compounds with similar binding modes to active compounds: (1) the generation of compounds with reasonable diversity reflected by their distribution in chemical space; and (2) the generation of novel molecules sharing similar binding modes with the classical active compounds. We also demonstrated the superiority of our model to a state-of-art 3D molecule generative model by testing them on three targets including hematopoietic progenitor kinase 1 (HPK1), SARS‐CoV‐2 main protease (3CL^pro^), and vitamin D receptor (VDR).

## Results

### Model design

**Figure 1:**
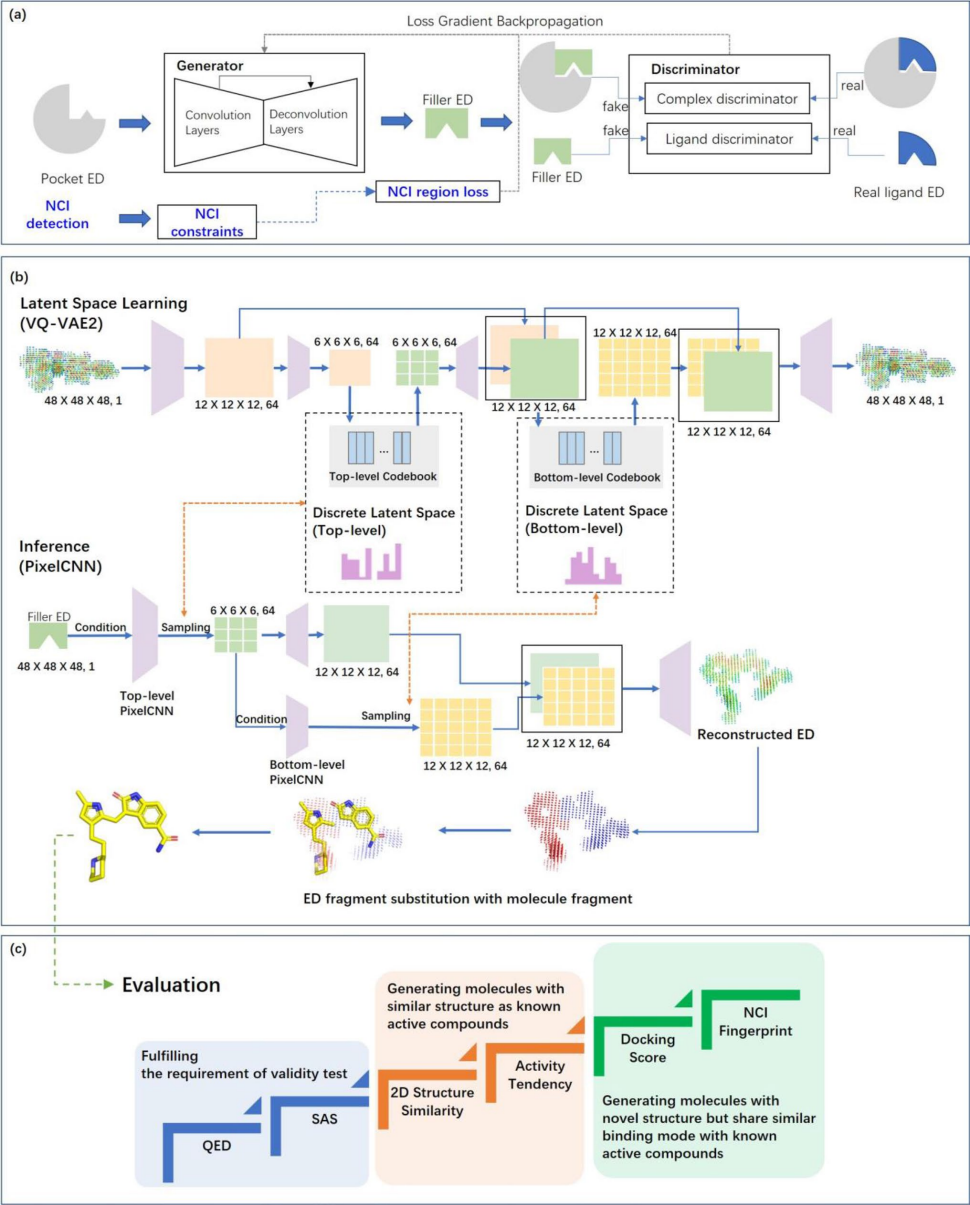
Model architecture. (**a**) The GAN for generating filler ED based on pocket ED. (**b**) ED interpretation module for molecule generation. VQ-VAE2 and PixelCNN used for latent space construction and autoregressive sampling, as well as the subsequent process of ED fragment substitution are illustrated. (**c**) The evaluation framework for generated molecules.

We trained our model to learn constraints and generate molecules in three major steps. Initially, a GAN was used to take the ED of a pocket as input to learn pocket-ligand complementarity and generate the ligand ED (Fig. 1a). We termed the generated ligand ED at this step as “filler ED” in this study. Subsequently, an ED interpretation module powered by vector quantized variational autoencoder (VQ-VAE2)^24^ was used to learn constraints on ligand validity and compress them in latent spaces. Then, PixelCNN^25^ was used to balance the complementary and molecular validity by sampling in the latent space using filler ED as conditions and thereby generate reconstructed ED (Fig. 1b). Such sampling process may produce multiple reconstructed EDs and thus achieve diversity. Next, reconstructed EDs were fragmented and thereby substituted with molecule fragments to generate final molecules. Experimental EDs were used to train the GAN, and quantum mechanics (QM) based computational ED as well as force field based molecular conformations were used to train VQ-VAE2 and PixelCNN. To assure the efficiency of retaining constraints during the training of GAN, we increased the weight of the region where the ligand and pocket show NCI. Details of the model design are provided in “Methods” section.

**Figure 2:**
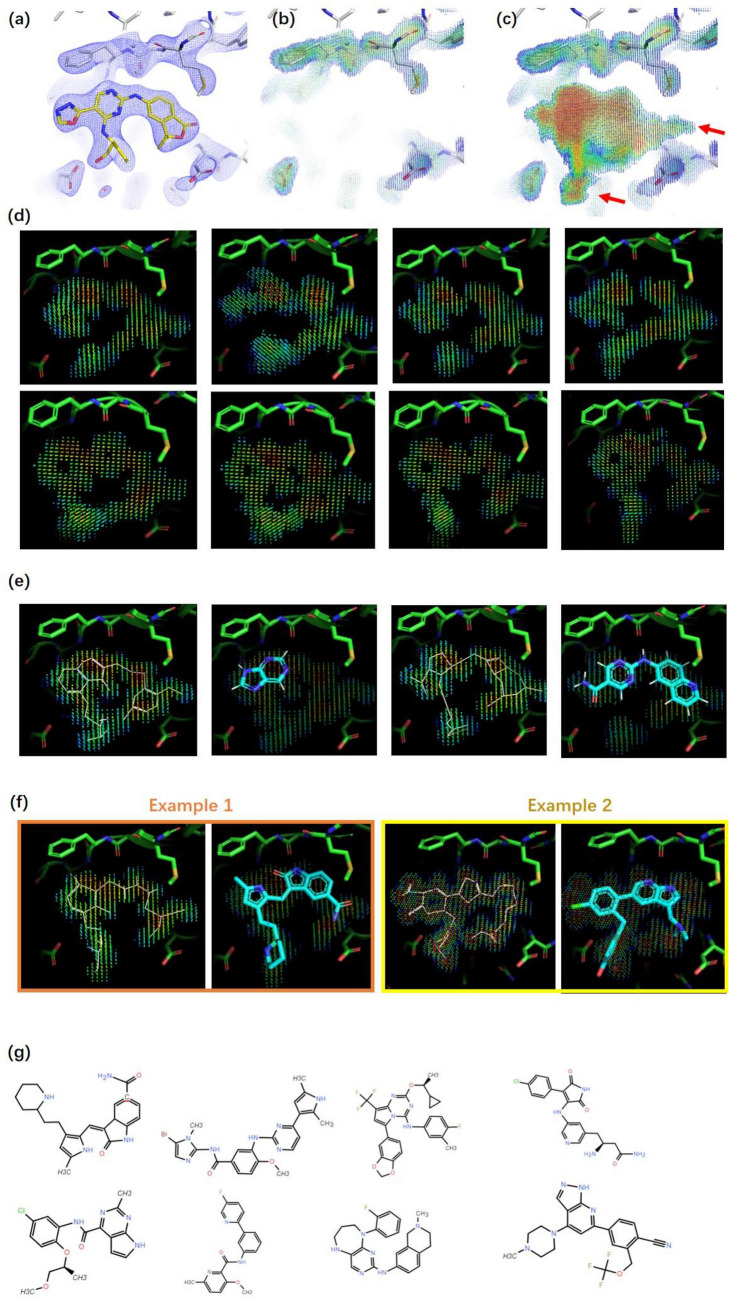
ED-based 3D molecule generation for HPK1. (**a**) Binding pocket and reference ligand (PDB code 7KAC). Experimental ED (2Fo-Fc map at 1.2 σ contour level) for the pocket and ligand is shown as blue mesh. (**b**) Pocket ED with ligand removed. (**c**) Generated filler ED. For the rainbow color scheme, red indicates a strong ED intensity, and blue indicates a weak ED intensity. The extension of generated ED to the region originally occupied by water molecules and cavities originally unoccupied is indicated by red arrows. (**d**) Reconstructed ED generated based on filler ED. (**e**) Hinge binding fragments fitted in the reconstructed ED; map skeletons shown as white lines. (**f**) Examples of generated molecules and their map skeletons aligned with reconstructed ED. (**g**) List of examples of generated molecules.

To demonstrate the working process of our model, we applied the model to a kinase target, HPK1. The complex structure of HPK1 binding with a reference ligand (PDB:7KAC; Fig. 2a) was used as a starting point. The pocket was defined as the residues within 5 Å from the reference ligand. Using experimental ED at a resolution of 2.5 Å for the pocket as input (Fig. 2b), the filler ED was generated as shown in Fig. 2c. Interestingly, the generated filler ED replaced the region originally occupied by water molecules and covered the unoccupied cavities (indicated with red arrows in Fig. 2c), demonstrating the complementarity of the generated filler ED to the pocket. Then, the generated filler ED was converted into several reconstructed EDs (Fig. 2d). For each reconstructed ED, a map skeleton was recognized to assist the fragmentation of reconstructed ED and the subsequent substitution of ED fragments with molecule fragments. For a kinase target, it is not a surprise to observe that the classical hinge binding groups could be well fitted into the reconstructed EDs (Fig. 2e). Finally, the molecular fragments were connected to create intact molecules (Fig. 2f,g). In the above process, the diversity of generated molecules could be achieved during reconstructed ED sampling and ED fragment substitution. On average, to generate one million molecules, over 2000 reconstructed EDs need to be sampled using the filler ED generated based on the pocket.

### Model evaluation

As shown in Fig. 1c, our molecule generative model was evaluated from three perspectives: (1) the ability to generate valid molecules in terms of QED and SAS while maintaining a reasonable diversity; (2) the ability to generate molecules similar to classical active compounds, defined as generation of molecules with over 0.5 Tanimoto similarity^8,26^ with the reference active compounds; (3) the ability to generate novel binders, defined as the generation of molecules with novel scaffolds relative to the reference active compounds but possessing similar binding modes.

We selected three targets including HPK1 (PDB: 7KAC), 3CL^pro^ (PDB:7VU6), and VDR (PDB: 1S19), to test the performance of our model. They were selected as representatives of kinase, protease, and nuclear receptors. Regarding references (Supporting information Table S1), 6334, 1101, and 757 compounds with reported activity were used for HPK1, 3CL^pro^, and VDR, respectively (Supplementary material 1). In addition, a state-of-art 3D molecule generative model^27^ reported in NeurIPS 2021 was used as a comparison benchmark.

### Validity test

We generated 10,000 molecules for each of the three targets using our model and the benchmark model and compared the QED and SAS of the generated molecules. To make the comparison results easy to understand, we also calculated the QED and SAS of the reference compounds and used them as positive controls. As shown in Table 1, although both our model and the benchmark model performed well for QED, our model outperformed the benchmark model on SAS.

**Table 1:**
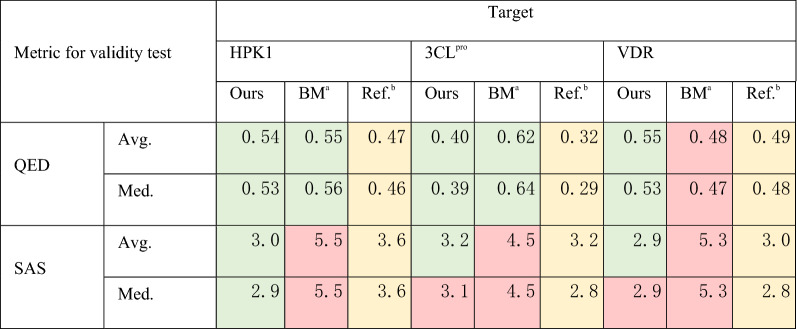
Evaluation of molecular validity.

10,000 molecules each were generated by our model and the benchmark model for each target; ^a^BM refers to a model^27^ reported in NeurIPS 2021 and used as the benchmark here; ^b^Reference compounds (Supporting information Table S1); Regarding the color scheme: all the values of reference compounds have their cells colored yellow. For generated molecules, if their values are better than or equal to that of the reference, then their cells are colored green; otherwise, their cells are colored red. Specifically, for QED, higher values are better; for SAS, lower values are better.

### Chemical space distribution

**Figure 3:**
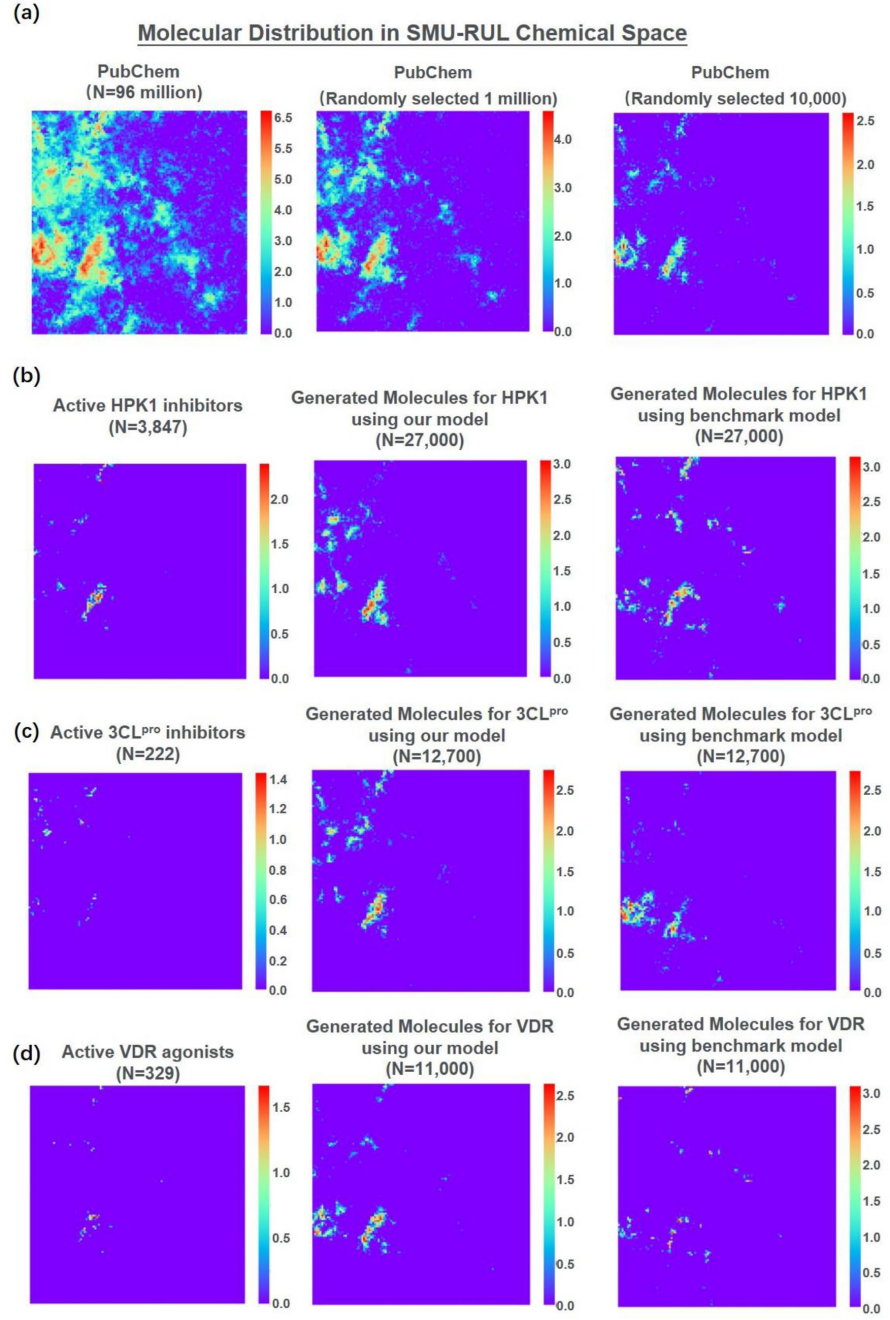
Chemical space distribution of generated molecules and references. A 120 × 120 SOM was created using SMU-RUL^28^ compounds. The color indicates the number of molecules on a logarithmic scale. The distributions of molecules from PubChem are listed in panel a. The distributions of reference active compounds and molecules generated using different models for HPK1, 3CL^pro^, and VDR are listed in panel (**b**–**d**), respectively. The number of molecules generated by our model is adjusted to match the number that can be generated by the benchmark model within a reasonable time frame. The SMILES of the molecules and their positions in the chemical space are provided in Supplementary material 3.

To understand the diversity and novelty of the molecules generated by our model, we calculated the Tanimoto-similarity-based diversity and compared the scaffold similarity of generated molecules with reference compounds. As shown in Table S2 and Figure S1 (Supporting information), the molecules generated by the benchmark model exhibit higher diversity and novelty than those generated by our model. However, such general diversity and novelty are not suitable for the evaluation of molecules generated with constraints, because well-functioning constraints may reduce the general diversity and novelty. To perform a comprehensive evaluation, we referred to the small-molecule-universe representative universal library (SMU-RUL) chemical space^28^, a self-organizing-map (SOM)-supported chemical space used to describe the distribution of molecules with molecular weight less than 500 Da. To simplify the comparison results, samples from PubChem were used to represent molecules lacking target-specific constraints, and active reference compounds were used to represent molecules with tight constraints. As shown in Fig. 3, we observed that our model achieved a better balance between the diversity and novelty and the constraints than the benchmark model (i.e., the molecules generated by our model are concentrated towards the region where the active compounds are positioned, whereas the molecules of the benchmark model are distracted toward some other regions and thus appear more diverse).

### Generation of molecules similar to classical active compounds

**Figure 4:**
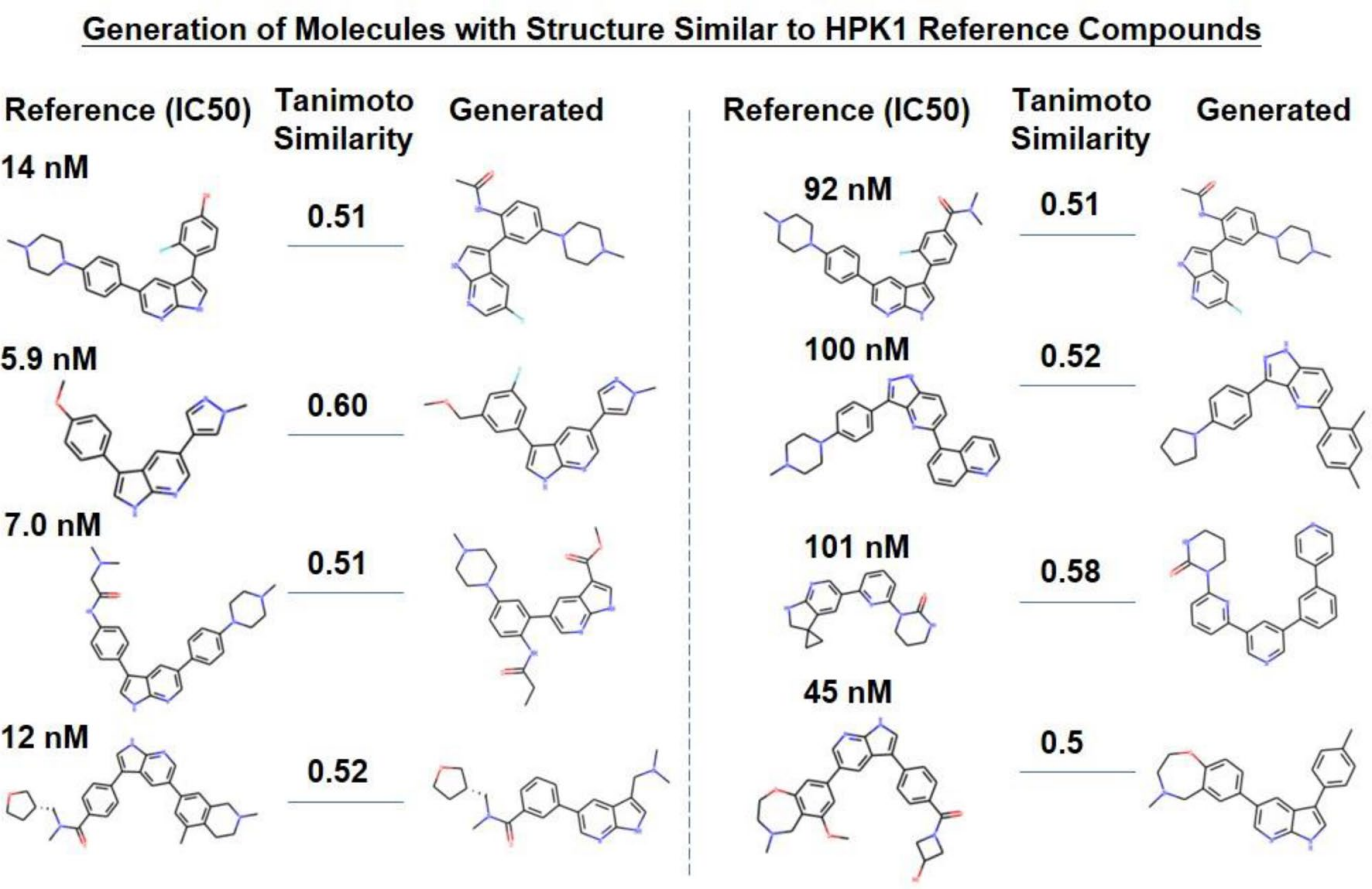
Examples of generated molecules that are similar to reference compounds for HPK1.

To test whether our molecular generative model can generate molecules with structures similar to already known active compounds, one million molecules were generated for each of the three targets and compared to reference compounds by measuring the Tanimoto similarity of the ECFP4 fingerprint. In this study, a molecule with over 0.5 Tanimoto similarity against a reference compound was considered a similar molecule to this reference compound. Our model successfully generated molecules similar to reference compounds for all three targets (Supplementary material 2). Taking HPK1 as an example, some reference compounds and their generated counterparts are listed in Fig. 4.

Furthermore, when we sorted reference compounds by their activity, we observed that our model tended to generate molecules with higher similarity to active and medium-active rather than inactive compounds, for all three targets (Table 2). Apart from the similarity trend, our model also generated more molecules similar to active and medium-active compounds than to inactive ones for HPK1 and 3CL^pro^. Regarding the comparison with the benchmark model which failed to generate million-level molecules within a reasonable time frame (Supporting information Table S3), it would be unfair to use all the 1 million molecules generated by our model. Therefore, our molecules that were subjected to the comparison were randomly selected from the 1 million previously generated to match the capacity of the benchmark model. As shown in Table S4 (Supporting information), although neither of the two models generated molecules similar to active reference compounds under the condition that only tens of thousands of molecules were generated, our model still provided molecules with better Tanimoto similarity to the references than the benchmark model did.

**Table 2:** Test for the generation of molecules similar to classical active compounds.

Metrics	HPK1			3CL^pro^			VDR		
	Active	Medium	Not active	Active	Medium	Not active	Active	Medium	Not active
# of reference compounds	3847	2319	168	222	248	631	329	67	361
# of molecules generated	1 million			1 million			1 million		
Max. of Tanimoto similarity to ref. cpd	0.76	0.72	0.51	0.58	0.73	0.61	0.57	0.44	0.55
# of reference molecule with similar^a^ counterparts generated	58	53	1	3	15	11	1	0	7
# of generated molecules similar to ref. cpd	30	20	2	14	158	42	1	0	10

^a^Similar: for a reference compound, if a generated molecule has over 0.5 Tanimoto similarity with it for ECFP4, then this reference compound is considered to have similar counterparts generated. The SMILES of the generated molecules and their similar reference compounds are provided in Supplementary material 2.

### Generation of novel compounds possessing similar binding modes as active compounds

If a molecule generative model can only generate classical active compounds, it will not be attractive to researchers searching for new drugs. To test our model’s ability to generate active compounds with novel structures, we searched the generated library for the molecules exhibiting a < 0.5 Tanimoto similarity with reference compounds while sharing a similar binding mode.

**Figure 5:**
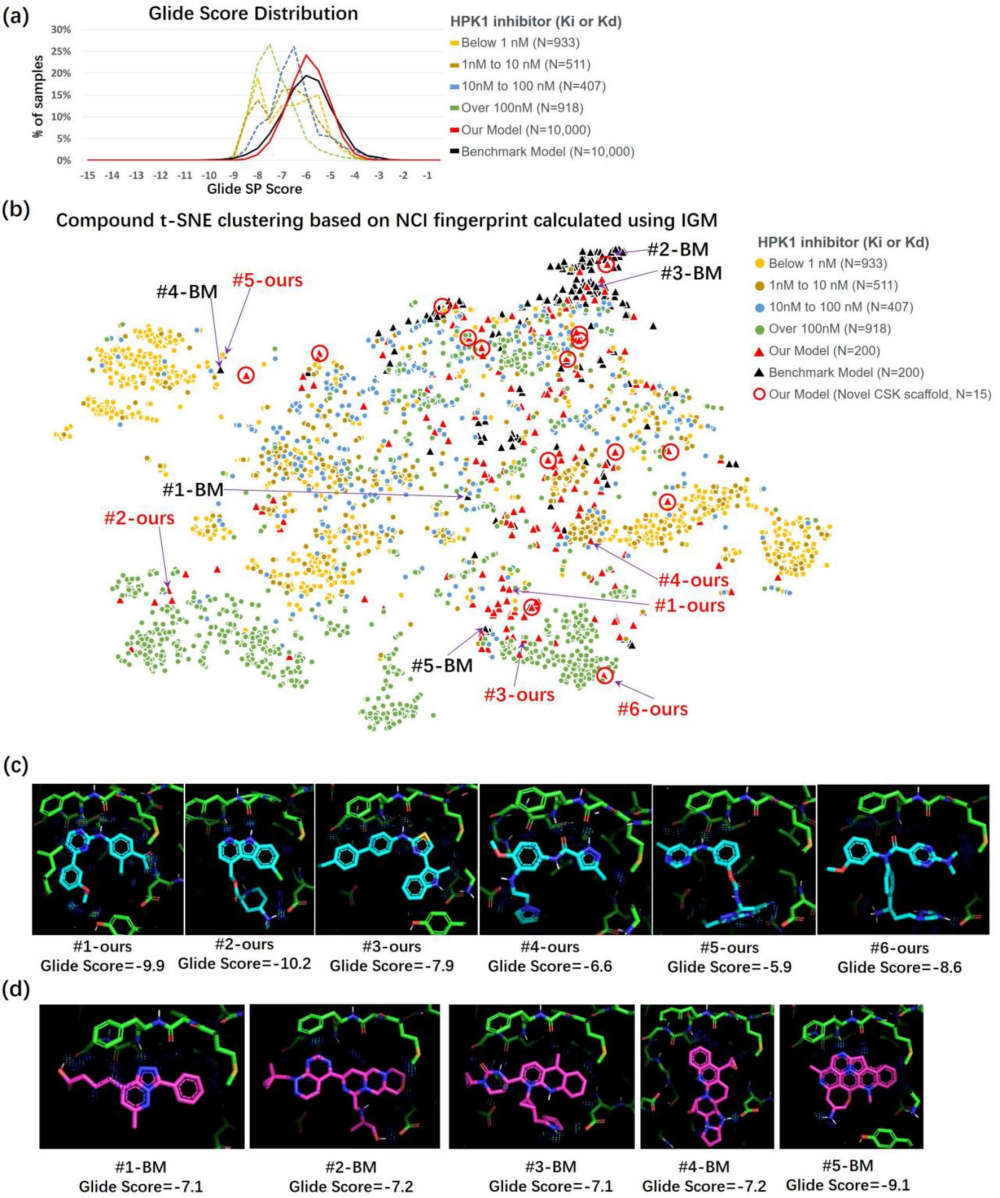
Binding mode analysis of the generated molecules for HPK1. (**a**) Glide Score distribution for active reference compounds and generated molecules. (**b**) Results of t-SNE clustering using IGM calculated NCIs as features. Red circles are used to indicate our generated molecules with novel cyclic skeletons^33^ (CSK) with respect to reference compounds. A generated molecule is defined as having novel CSK if the highest Tanimoto similarity between its CSK and that of all the reference compounds is less than 0.5. (**c**) Binding mode of selected molecules generated by our model. (**d**) Binding mode of selected molecules generated by benchmark model. For panel c and d, NCI regions are indicated with dots colored using the rainbow scheme, in which blue indicates weak interactions and red indicates strong interactions. More details are provided in Supplementary material 4, 5, and 6.

Taking HPK1 as an example, 2769 HPK1 reference compounds with reported Ki or Kd were selected and docked into the 7KAC pocket by using Glide SP^29,30^ to provide a background for binding mode analysis. This is because the binding mode is more theoretically related to Ki and Kd than to EC50 or IC50. However, as shown in Fig. 5a, the Glide score could not efficiently distinguish the reference compounds with different activities, which implies the need for a more powerful descriptor of binding mode. Thus, we referred to NCI fingerprints depicted using the independent gradient model (IGM) method^31^. The IGM method calculates NCIs by analyzing the topological properties of EDs, and thus can provide a full spectrum of NCIs^13^, whereas the traditional rule-based method only provides a short list of classical NCIs. Compound clustering based on the NCI fingerprint was conducted using T-distributed stochastic neighbor embedding (t-SNE)^32^. As shown in Fig. 5b, the clustering results aligned quite well with the activity: compounds sorted into one cluster tended to possess similar activities despite the low discriminative capability for samples with very high activity (separating compounds below 1 nM from those below 10 nM).

To analyze the binding mode of molecules generated for HPK1, we randomly selected 10,000 generated molecules with < 0.5 Tanimoto similarity against the reference compounds and then scored them using Glide by only employing the minimization and scoring functions. This operation was conducted for both our model and the benchmark model. Similarly to the reference compounds, the Glide score was unable to efficiently distinguish between the performances of our model and the benchmark model (Fig. 5a). Therefore, we randomly selected 200 molecules from each of the two models and conducted NCI fingerprint clustering. As shown in Fig. 5b, the molecules generated by the benchmark model tended to be more concentrated and farther from the cluster of active compounds than those generated by our model. Molecules at different positions on the NCI fingerprint map are listed in Fig. 5c,d, and their NCI fingerprints are provided in Fig. S2 (Supporting information). The hinge region-related NCIs, which are considered crucial for active compounds, were exhibited for molecules generated close to the cluster of active compounds. These NCIs were weak for molecules far from the clusters, such as #2-BM and #3-BM. Although there were cases (#4-BM and #5-ours) where the molecule from our model was close to the benchmark model molecule in the NCI fingerprint map, and both of them were close to the cluster of active compounds, our molecules are still easily distinguished for their superior synthetic accessibility.

## Discussion

Our model is designed under the principle of learning constraints in separated phases: in phase I, less abundant experimental ED data supports the GAN to learn pocket-ligand complementarity, which is reflected by filler ED; in phase II, relatively abundant computational data support the VQ-VAE2 to learn the constraints on molecule validity, which is reflected by latent space. To balance the constraints learned in different phases, an autoregressive sampling process (i.e., PixelCNN) is designed, in which phase I generated filler ED is used as conditions to sample in phase II generated latent space. Such sampling has the additional advantage of diversifying the generated molecules. In summary, because an optimal filler fulfilling all the NCIs with a pocket may not exist as a valid druglike molecule, the above design makes it possible to first focus the use of high-value experimental data in a single task and then balance the constraints learned from different tasks.

However, the above design also raises additional challenges, such as the evaluation of ED and the efficiency of inheriting constraints such as NCI in different tasks. To address these challenges, we again refer to the concept of “map skeleton” which is previously used to assist the fragmentation of reconstrued ED. Because one reconstructed ED has only one map skeleton and the nodes of a map skeleton represent the most possible positions where atoms locate, whether a map skeleton of an ED is druglike and capable of forming NCI indicate the quality of that ED. Specifically, the following indices can be used for ED quality evaluation: (1) what percentage of contacts between filler ED and solvent accessible hetero atoms (SAHA) of the pocket is retained in reconstructed EDs and map skeletons; (2) whether a map skeleton can be interpreted into valid SMILES; (3) whether a map skeleton contains rings. Taking 2500 reconstructed EDs generated by our model as example, the reconstructed EDs and map skeletons retain around 100% (e.g., 82%/82% for HPK1) and 70% (e.g., 59%/82% for HPK1) of the contacts between filler ED and pocket SAHA (Supporting information Fig. S3), respectively. To check from the perspective of drug likeness, 97% and 80% of the reconstructed EDs have map skeletons with at least one ring and as valid SMILES, respectively.

To further improve the performance of our model, there are two major points under consideration. First, we are considering the use of multi-resolution ED for the GAN training. Currently, the pockets used for training were represented as ED at a resolution of 2.5 Å, and therefore, some of the PDB entries with a resolution higher than 2.5 Å had their data at high-resolution shells unused. One possible method for utilizing these valuable data is to create multiple channels for different resolutions. However, one must strike a balance between creating multiple channels and avoiding the data sparsity problem for high-resolution channels receiving PDB entries with low-resolution data. Second, the fragment substitution approach employed in this study is to demonstrate the ED-based idea and thus has a relatively simplified design whose performance is highly related to the quality of the fragment library. To upgrade the approach, we can train a clustering model to cut the ED into more fragments with smaller size than the current approach does. Doing so will reduce the size of the fragment library to be searched, as the number of conformations decreases sharply along with the shrink of the size of molecule fragments. In addition, we are considering expending the training set of VQ-VAE2 to cover all eight million molecules in SMU-RUL and more molecules from PubChem. Doing so will improve the quality of latent space and reconstructed ED so that the subsequent V-Net may directly produce high-quality molecules instead of map skeletons, making the fragments assembling unnecessary. Another approach worth trying is to generate a caption in the format of SMILES that best describes the reconstructed ED. Then, the generated SMILES can be fitted in the reconstructed ED to get 3D conformation.

Another point that should be considered is how to use our model in a scenario in which there is no experimental ED data as input. Such a scenario could occur when the generative model is used for a pocket provided by a molecular dynamic approach. Our model, although trained using experimental ED data as input, also works when using a calculated Fourier synthesis-based ED map as input. Specifically, one only needs to add reasonable B factors (e.g., 20 with a Gaussian perturbation) for each atom and then calculate the ED map using the Fourier synthesis. Furthermore, the application of our molecule generative model to the representative conformations obtained from molecular dynamics may provide potential solutions for some challenging tasks. First, it is possible to remove the bias introduced by using only one conformation of the pocket. We can generate molecules for all the representative conformations and all the available experimental EDs for the pocket (if any), and then combine the generated molecules to compile the library. Second, it is possible to support cosolvent MD simulations (CMD) in the study of allosteric pockets and hidden pockets^34,35^. Because such pockets usually require the presence of a ligand to open from an apo state, the incorporation of the right cosolvents is highly associated with the success of sampling the open state. Usually, general probes such as benzene and isopropanol are used as probes^34^. With our model, specific probes can be generated for the cavity of interest. In addition, if the cavity exhibits conformational changes after the probe binds to it during CMD, we can use the new conformation of the cavity to generate new probes.

Another potential use of our model is the detection of small molecule binding regions for protein–protein interaction (PPI) interfaces. Because an electron density map can represent the physical and chemical properties of the molecule and is continuously smoothing, it can fully utilize the potential of CNN in representing the local environment. Specifically, although we did not train our model with PPI samples, our model can still recognize the “pocket-like” region in PPI interfaces and generate fragment-sized molecules (Supporting information Figure S5).

## Methods

### Molecule design

#### Ligand ED generative model

As show in Fig. 1a, the pocket EDs were input into a V-Net 3D image generation network, which acts as a generator. In addition to the generator, two discriminators, called complex and ligand discriminators, were designed. The complex discriminator was responsible for examining the level of complementarity between the ligand and the pocket, and the ligand discriminator was responsible for verifying ED validity from the perspectives of gradient and connectivity.

The protein-ligand pairs used to train the filler ED generative model were from the PDBbind database^36^. In total, 27,006 ligand-protein pairs were extracted from 12,905 complexes. The number of the ligand-protein pairs was larger than that of the complexes because we included drug-like molecules as well as other binders with molecular weight less than 600, such as ATP, ADP, and sugars. To avoid the appearance of similar pockets in both the training and the testing sets, training-testing split was done by referring to the pocket classification from previous studies focusing on the 1D and 3D pocket similarity^11,37^. The key NCIs identified using a previously reported method^13^ were also included in the model during training with the purpose of making the network more attentive on the NCI related regions. Smooth L1 loss was performed separately to strengthen the loss of the NCI related regions.

The GAN and its loss were expressed using the following equations:

1 $${G}^{*}=argmi{n}_{G}ma{x}_{D}\left({L}_{cGAN}\left(G,{D}_{complex}\right)+\alpha {L}_{GAN}\left(G,{D}_{ligand}\right)\right)+\lambda L\left(G\right)$$

2 $${L}_{cGAN}\left(G,{D}_{complex}\right)={\mathbb{E}}_{x,y}\left[log{ D}_{complex}\left(x,y\right)\right]+{\mathbb{E}}_{x,y}\left[\mathrm{log}\left(1-{D}_{complex}\left(x,G\left(x\right)\right)\right)\right]$$

3 $${L}_{GAN}\left(G,{D}_{ligand}\right)={\mathbb{E}}_{x,y}\left[log{ D}_{ligand}\left(x,y\right)\right]+{\mathbb{E}}_{x,y}\left[\mathrm{log}\left(1-{D}_{ligand}\left(G\left(x\right)\right)\right)\right]$$

4 $$L\left(G\right)= \beta {L}_{gen}+\gamma {L}_{ligand}+ \varepsilon {L}_{NCI}+\delta {(L}_{tv1}+ {L}_{tv2})$$

where *D*_*complex*_ represents the complex discriminator, and *D*_*ligand*_ represents the ligand discriminator. *L(G)* indicates the regression loss of GAN, *x* represents the input pocket*, y* represents the ground truth ligand, and *α*, *λ*, *β*, *γ*, *ε*, $\delta$ are hyper parameters. *L*_*tv1*_ and *L*_*tv2*_ are used to measure the similarity of two EDs from the perspectives of the first and second orders derivative of intensity, respectively. The addition of *D*_*ligand*_ and the incorporation of *L*_*tv1*_ and *L*_*tv2*_ in *L*(*G*) are to ensure the learning of ED topology properties which reflect not only the geometrical information such as molecule shape but also chemical information such as atom type.

The components of *L*(*G*) are defined as follows:

5 $$\mathrm{SmoothL}1=\left\{\begin{array}{l}0.5{\mathrm{x}}^{2},\quad if \left|\mathrm{x}\right|<1\\ \left|\mathrm{x}\right|-0.5,\quad otherwise\end{array}\right.$$

6 $${L}_{gen}=\frac{1}{{N}_{I}}\sum_{\mathrm{i}\in \mathrm{I}}\mathrm{SmoothL}1({G(x)}_{i}-{y}_{i})$$

where *I* indicates the entire region, and *N*_*I*_ indicates the number of elements in $I$.

7 $${L}_{ligand}=\frac{1}{{N}_{Gt}}\sum_{\mathrm{i}\in \mathrm{Gt}}\mathrm{SmoothL}1({G(x)}_{i}-{y}_{i})$$

where *Gt* indicates the region covered by the ground truth, and *N*_*Gt*_ indicates the number of elements in *Gt*

8a $${L}_{NCI}=\frac{1}{{N}_{NCI}}\sum_{\mathrm{i}\in \mathrm{NCI}}\mathrm{SmoothL}1({G(x)}_{i}-{y}_{i})$$

where *NCI* indicates the region covered by *NCI*, and *N*_*NCI*_ indicates number of elements in *NCI*

8b $${L}_{tv1}=\frac{1}{{N}_{I}}\sum_{\mathrm{i}\in \mathrm{I}}\mathrm{SmoothL}1({\widehat{tv1}}_{i}-{tv1}_{i})$$

8c $${L}_{tv2}=\frac{1}{{N}_{I}}\sum_{\mathrm{i}\in \mathrm{I}}\mathrm{SmoothL}1({\widehat{tv2}}_{i}-{tv2}_{i})$$

8d $$tv1= \left({I}_{\left(i+1,j,k\right)\in I}-{I}_{\left(i,j,k\right)\in I}\right) \oplus \left({I}_{\left(i,j+1,k\right)\in I}-{I}_{\left(i,j,k\right)\in I}\right) \oplus \left({I}_{\left(i,j,k+1\right)\in I}-{I}_{\left(i,j,k\right)\in I}\right)$$

8e $$tv2= \left({tv1}_{\left(i+1,j,k\right)\in I}-{tv1}_{\left(i,j,k\right)\in I}\right) \oplus \left({tv1}_{\left(i,j+1,k\right)\in I}-{tv1}_{\left(i,j,k\right)\in I}\right) \oplus \left({tv1}_{\left(i,j,k+1\right)\in I}-{tv1}_{\left(i,j,k\right)\in I}\right)$$

where $\oplus$ indicates the operator of concatenate.

To prepare data for model training, the experimental EDs (i.e. sigma scaled 2Fo-Fc map) for pockets were generated using Phenix^38^ and experimental ED coefficients downloaded from PDB. These pocket EDs were used as input features for the AI model. The computational ED for ligands were prepared as labels. The software xtb^39^ were used to calculate computational ED for ligands at GNF2-xTB level using ligand coordinates as input. Since the ED intensity within the nuclear region is much stronger than that within the region where the NCIs take place and where the shape of the molecule is defined (i.e., the isosurface at 0.03 e/Å^3^), the range of values were compressed using logarithms and then used as labels for the AI model.

ED was featured using 3D CNN, and data augmentation was used to achieve rotational invariance.

#### Ligand structure generative model

As shown in Fig. 1b, VQ-VAE2 was used to compress the ED into a discrete latent space and learn a codebook in which EDs are represented by a series of embeddings. To retain the constraints in the input ED while ensuring that the output ED can match a valid molecule, a separate autoregressive prior (PixelCNN) was taught to sample the latent space with input ED as a given condition. Two codebooks were learned during training: the top-level codebook focusing on the extraction of general profile information (such as shape) and the bottom-level codebook focusing on the detailed information (such as local conformations). Regarding sampling with the two codebooks, the input ED was used as a condition to sample the top-level codebook, and the top-level encoding was used as the condition to sample the bottom-level codebook. Thus, the relationship between the conditional ED (i.e., input ED) and the generated ED was decoupled.

EDs were voxelized into a discrete 0.5 Å cubic grid with a side size of 24 Å. There were represented as a tensor $\mathrm{p}\in {\mathcal{R}}^{1\times 48\times 48\times 48}$, where channel size was 1, expressing the intensity of ED. The VQ-VAE2 loss is shown in Eq. (9),where *x* is the training instance, *D* is the decoder of the VQ-VAE2 and *e* is the encoder. The reconstruction loss is shown in Eq. (10). It combines the ${\mathrm{L}}_{\mathrm{SoftDiceLoss}}$ as well as ${\mathrm{L}}_{\mathrm{SmoothL}1}$. To generate better ED reconstructed shapes, we adopted ${\mathrm{L}}_{\mathrm{SoftDiceLoss}}$ as shown in Eq. (11) for segmenting ED and the background where *y* is the target and $\widehat{y}$ is the prediction. The target label of the pixel with the ED value was set to 1, and the rest of the background part was set to 0. Further, ${\mathrm{L}}_{\mathrm{SmoothL}1}$ was obtained using Eq. (12); similarly, y is the target and $\widehat{\mathrm{y}}$ is the prediction.

9 $$\mathrm{L}(\mathrm{x},\mathrm{D}(\mathrm{e})) = {\mathrm{L}}_{\mathrm{Reconstruction}}(\mathrm{x},\mathrm{D}(\mathrm{e})) + {\Vert \mathrm{sg}[\mathrm{E}(\mathrm{x})-\mathrm{e}]\Vert }_{2}^{2}+\upbeta {\Vert \mathrm{sg}[\mathrm{e}]-\mathrm{E}(\mathrm{x})\Vert }_{2}^{2}$$

10 $${\mathrm{L}}_{\mathrm{Reconstruction}}(\mathrm{x},\mathrm{D}(\mathrm{x})) = {\mathrm{L}}_{\mathrm{SoftDiceLoss}}(\mathrm{x},\mathrm{D}(\mathrm{x}))+\mathrm{\alpha }{\mathrm{L}}_{\mathrm{SmoothL}1}(\mathrm{x},\mathrm{D}(\mathrm{x}))$$

11 $${\mathrm{L}}_{\mathrm{SoftDiceLoss}}(\widehat{\mathrm{y}},\mathrm{y}) = 1 - \frac{2\sum_{\mathrm{i}\in \mathrm{I}-1}{\widehat{\mathrm{y}}}_{\mathrm{i}}{\mathrm{y}}_{\mathrm{i}}}{\sum_{\mathrm{i}\in \mathrm{I}-1}{{\widehat{\mathrm{y}}}_{\mathrm{i}}}^{2}+\sum_{\mathrm{i}\in \mathrm{I}-1}{{\mathrm{y}}_{\mathrm{i}}}^{2}}$$

12 $${\mathrm{L}}_{\mathrm{SmoothL}1}(\widehat{\mathrm{y}},\mathrm{y})= \frac{1}{{N}_{I}}\sum_{\mathrm{i}\in \mathrm{I}}\mathrm{SmoothL}1({\widehat{y}}_{i}-{y}_{i})$$

*PixelCNN* optimized the negative log-likelihood of the training data *to maximize the probability* $\mathrm{p}(\mathrm{x}|\mathrm{h}) = \prod_{\mathrm{i}=1}^{\mathrm{HWD}}\mathrm{p}({\mathrm{x}}_{\mathrm{i}}|{\mathrm{x}}_{1},...,{\mathrm{x}}_{\mathrm{i}-1},\mathrm{h})$, where H represents height, W represents width, and D represents depth of the 3D cube where h is the condition. Top-level and bottom-level prior networks were modeled with 6 × 6 × 6 and 12 × 12 × 12 latent variables, respectively. Additionally, the condition of the top-level PixelCNN was the previously generated filler ED. The bottom-level network was conditioned on the top-level prior.

To prepare the data for modeling training, two million molecules with a QED exceeding 0.3 were extracted. For each molecule, 20 conformers were generated using ConfGen^40^. EDs of the 40 million conformers were generated using xtb^39^ and then used as training data for the VQ-VAE2.

#### Map skeleton assisted ED fragment substitution with molecule fragment

First, the reconstructed ED was submitted to a V-Net framework for atom detection. This step is similar to that of key point detection in human skeletons in the field of image processing. The V-Net was trained using the above 40 million EDs as features and the conformers used to generate these EDs as labels. The ground truth was represented as a tensor, where 10 channels were denoted based on atom type: PAD (no atoms), C, N, O, S, P, F, Cl, Br, and I. The cross-entropy loss was used for atom type classification. The V-Net output was comprised of connected atoms and called a map skeleton.

Then, a molecular fragment library was prepared. The molecules from PubChem were matched with 35 reaction templates^41^ according to the substructure superposition supported by RDKit^42^, and then cut into two fragments at the matching site. If a molecule matched multiple reaction templates, it was still cut into two fragments each time, but the cutting site was different. In this way, over 270,000 fragments with labeled cutting sites were obtained. Then OpenBabel^43^ was used to generate a maximum of 20 conformations for each fragment, and it finally produced a library with 3.5 million 3D conformations. GFN0-xtb EDs of the conformations were calculated for future use.

Next, the map skeleton was cut into several fragment pairs in the following manner: each time the whole molecule was cleaved at one acyclic single bond, two fragments were generated; next time, the whole molecule was cleaved at another acyclic single bond; the process was repeated until all acyclic single bonds had been cut. Each cut on the map skeleton generates a pair of map skeleton fragments and a pair of ED fragments. The above-established 3.5 million 3D conformation library was searched for entries that could be well fitted in the ED fragment in terms of shape and intensity and similar to the map skeleton fragment in terms of atomic position. Specifically, an entry in the library was first superimposed with the map skeleton fragment by minimizing root-mean-square deviation of atomic positions and then subjected to the measuring of Dice using Eq. (11) between its ED and the ED fragment. Next, the two groups of selected entries were assembled in an enumerative way to make a list of target molecules. Subsequently, several filters were applied to remove the unqualified molecules. These filters include the following:

1. Collisions with the pocket : the distance from the pocket heavy atom is less than 2.5 Å.
2. Collisions between the fragments: the distance from the non-bonded heavy atom is less than 2.0 Å.
3. Stability and synthetic accessibility filters and drug-like filter reported by Virshup et al.^28^

Illustrations can be found in Figure S4 and Table S5 in Supporting Information.

### Model evaluation

The data used for chemical space construction consisted of 8.8 million molecules from SMU-RUL^28^.

The evaluation data were collected from 144 publications including research papers and patents.

### Chemical space construction

Chemical space was constructed mainly based on the method described in a previous study^28^. In our work, four properties including carbon-scaled atomic mass, carbon-scaled atomic van der Waals volume, carbon-scaled atomic Sanderson electronegativity, and carbon-scaled atomic polarizability were used to calculate the autocorrelation fingerprint. The autocorrelation descriptors were calculated using PyBioMed^44^.

The SOM^45^ with size of 120 ×120 was implemented using MiniSom library^46^.

### NCI fingerprint analysis

Docking was implemented by using Glide^29,30^. IGM-based NCI analysis was implemented using Multiwfn^47^. The atom-pair-based NCI list output by Multwfn was further annotated with the Mol2/Sybyl atom types for both of the atoms using OpenBabel^48^ and PyBel^43^ packages, and then submitted to Scikit-learn^49^ for t-SNE^32^ clustering analysis.

### Benchmark model

The source code of benchmark model is downloaded from its official website: https://github.com/luost26/3D-Generative-SBDD.

## Supplementary Information

Supplementary Information 1.

Supplementary Information 2.

Supplementary Information 3.

Supplementary Information 4.

Supplementary Information 5.

Supplementary Information 6.

Supplementary Information 7.

## Data Availability

The data analyzed during this study are included in this published article and its supplementary information files. Partial codes are available from the corresponding author on reasonable request. Our model provides service to academic users via https://edmg.stonewise.cn/#/create.

## Abbreviations

ED: Electron density
GAN: Generative adversarial network
NCI: Non-covalent interactions
QED: Quantitative estimate of drug-likeness
SAS: Synthetic accessibility score
VQ-VAE: Vector quantized variational autoencoder
t-SNE: T-distributed stochastic neighbor embedding
SOM: Self-organizing-map
SMU-RUL: Small-molecule-universe representative universal library
HPK1: Hematopoietic progenitor kinase 1
3CL^pro^: SARS‐CoV‐2 main protease
VDR: Vitamin D receptor
