# Supplementary material for: A pocket-based 3D molecule generative model fueled by experimental electron density
Source: Sci Rep. 2022 Sep 6;12:15100. doi: 10.1038/s41598-022-19363-6 (PMC9448726; doi:10.1038/s41598-022-19363-6)
Supplement: Supplementary file 7 — Supplementary Information 7. [file 41598_2022_19363_MOESM7_ESM.docx]

Supporting Information

A pocket-based 3D molecule generative model fueled by experimental electron density

Lvwei Wang **^†1^**, Rong Bai**^†1^**, Xiaoxuan Shi**^†1^**, Wei Zhang**^2^**, Yinuo Cui**^1^**, Xiaoman Wang**^1^**, Cheng Wang**^1^**, Haoyu Chang**^1^**, Yingsheng Zhang**^1^**, Jielong Zhou**^1^**, Wei Peng**^2^**, Wenbiao Zhou**^1^**, and Bo Huang**^1^**

1. Beijing StoneWise Technology Co Ltd., Haidian Street #15, Haidian District, Beijing 100080, China
2. Innovation Center for Pathogen Research, Guangzhou Laboratory, Guangzhou 510320, China

**List of Supplementary Tables and Figures**

**Table S1. Reference compounds**

**Table S2. Diversity of generated molecules**

**Table S3. Speed comparison**

**Table S4. Comparison with benchmark model in generating molecules similar to reference compounds**

**Table S5. Further examples of the molecular fragment assembly illustrated in Figure S4**

**Figure S1. Scaffold novelty analysis**

**Figure S2. NCI fingerprint of selected molecules**

**Figure S3. Inheritance of contacts with the solvent accessible hetero atoms (SAHA) of the pocket through different tasks**

**Figure S4. Illustration of molecular fragment assembly**

**Figure S5. Recognizing small molecule binding regions in PPI interfaces**

**Table S1. Reference Compounds**

| **Target** | **Number of reference compounds** | | | |
| --- | --- | --- | --- | --- |
|  | **Active** | **Medium** | **Not Active** | **Total** |
| HPK1 | 3,847 | 2,319 | 168 | 6,334 |
| 3CL^pro^ | 222 | 248 | 631 | 1,101 |
| VDR | 329 | 67 | 361 | 757 |

Note: Active = < 100 nM potency (Ki, Kd, EC50, or IC50); Medium = 100–1,000 nM potency; Not active = > 1,000 nM potency

**Table S2. Diversity of Generated Molecules**

| Diversity | | Target | | | | | | | | |
| --- | --- | --- | --- | --- | --- | --- | --- | --- | --- | --- |
|  |  | HPK1 | | | 3CL^pro^ | | | VDR | | |
|  |  | Ours | BM^a^ | Ref.^b^ | Ours | BM^a^ | Ref.^b^ | Ours | BM^a^ | Ref.^b^ |
| Diversity^a^ | Avg. | 0.86 | 0.89 | 0.82 | 0.86 | 0.90 | 0.68 | 0.86 | 0.86 | 0.64 |
|  | Med. | 0.87 | 0.90 | 0.85 | 0.86 | 0.91 | 0.84 | 0.87 | 0.86 | 0.54 |

**Note:** ^a^Diversity = 1- (the average of pairwise Tanimoto similarities over ECFP4 fingerprints among the molecules). The number of reference molecules subjected to the analysis is the same as that of Table 1. For each target, 10,000 molecules were generated and analyzed.

**Table S3. Speed Comparison**

|  | Number of Unique Molecules Generated in 12 hours^*^ | | |
| --- | --- | --- | --- |
|  | HPK1 | 3CL^pro^ | VDR |
| Our Model | 1,142,718 | 265,152 | 560,328 |
| Benchmark Model | 2,395 | 4,170 | 1,965 |

Note: *With one NVIDIA*^®^*GeForce*^®^*RTX 2080 Ti

**Table S4. Comparison with Benchmark Model in Reproducing Reference Compounds**

| Metric for classical active compounds reproduction test | | **HPK1** | | | **3CL^pro^** | | | **VDR** | | |
| --- | --- | --- | --- | --- | --- | --- | --- | --- | --- | --- |
|  |  | Active | Medium | Not active | Active | Medium | Not active | Active | Medium | Not active |
| # of reference compounds | | 3847 | 2319 | 168 | 222 | 248 | 631 | 329 | 67 | 361 |
| Our Model | # of molecules generated | **27,000** | | | **12,700** | | | **11,000** | | |
|  | Max. of Tanimoto similarity to ref. cpd. | 0.49 | 0.47 | 0.44 | 0.38 | 0.50 | 0.43 | 0.42 | 0.42 | 0.50 |
| Benchmark Model | # of molecules generated | **27,000** | | | **12,700** | | | **11,000** | | |
|  | Max. of Tanimoto similarity to ref. cpd. | 0.36 | 0.37 | 0.35 | 0.34 | 0.47 | 0.52 | 0.31 | 0.33 | 0.4 |

**Table S5. Further Examples of the Molecular Fragment Assembly Illustrated in Figure S4**

| **Fragments Group #** | **SMILES (cutting site indicated by *)** | **Leaving Group** | **Reaction Template** |
| --- | --- | --- | --- |
| 1 | *Nc1cc(C(=O)NCc2cccnc2)ccc1Cl | H | alkylation-1 |
|  | *C#CCOC(=O)NCCCC | I | alkylation-1 |
| 2 | *Oc1cc(C(=O)Nc2cccnc2)ncc1OC | H | ether-2 |
|  | *CCCOc1ccc(Cl)cc1 | Br | ether-2 |
| 3 | *Nc1cc(C(=O)Nc2cc(C)ccn2)ccc1C | H | alkylation-1 |
|  | *CCCOc1ccc([N+](=O)[O-])cc1 | Br | alkylation-1 |
| 4 | *Nc1cc(C(=O)Nc2cc(C)ccn2)ccc1C | H | alkylation-1 |
|  | *CCCOc1ccc(F)cc1 | Cl | alkylation-1 |

**
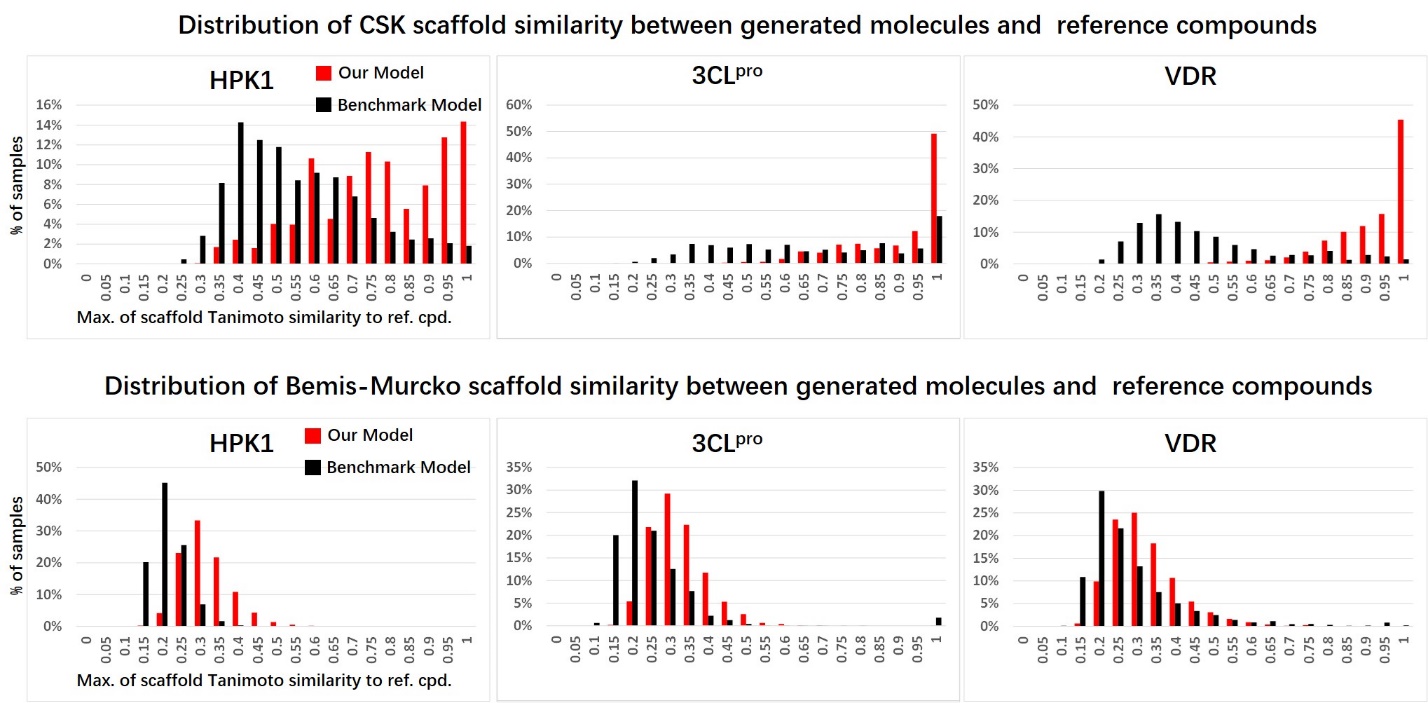
**

Figure S1. Scaffold novelty analysis. Distributions of scaffold similarity between generated molecules and reference compounds. For each target, 10,000 molecules respectively generated using our model and the benchmark model were subjected to analysis. The scaffold similarity is measured by using Tanimoto coefficient.

*
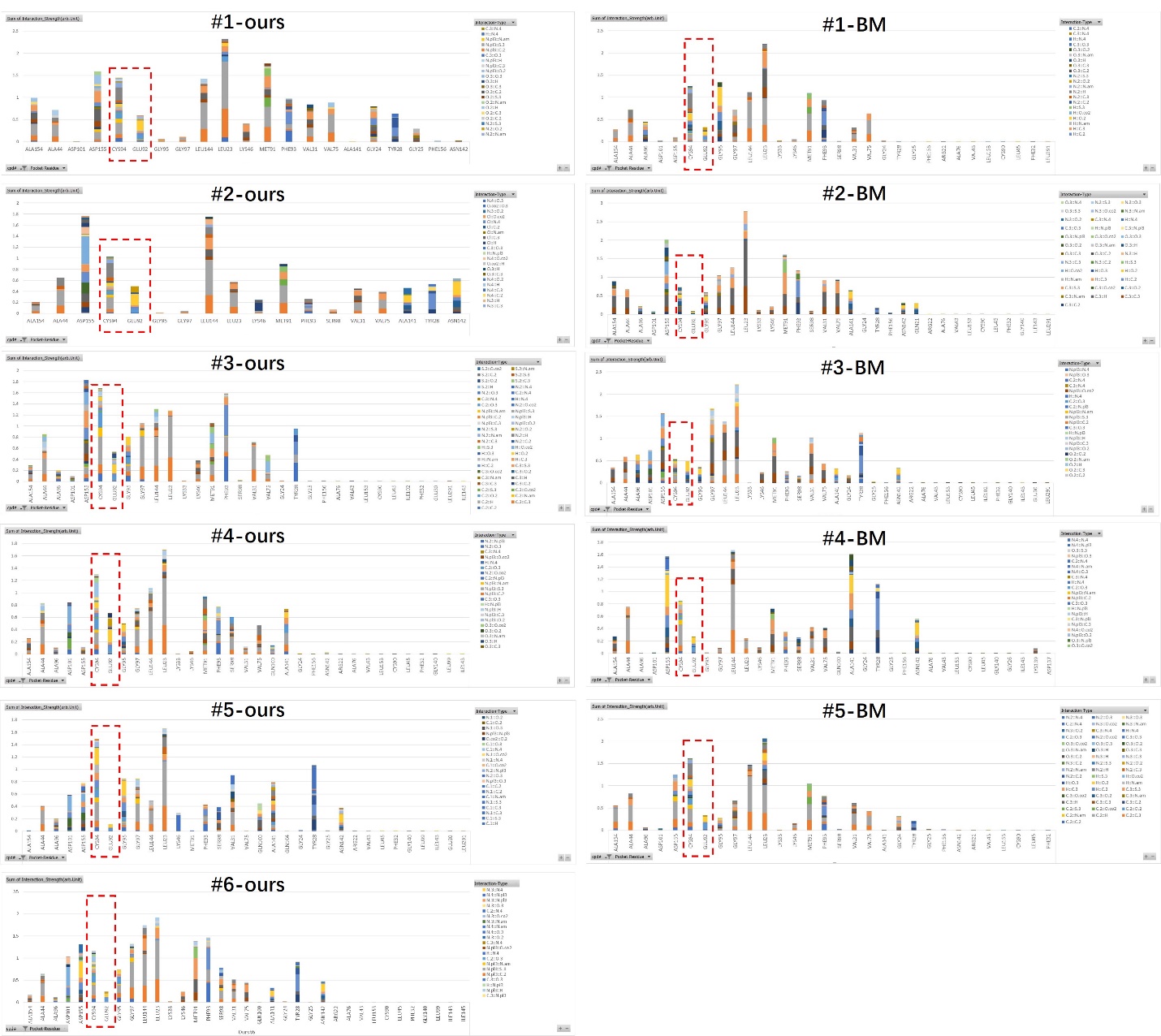
*

Figure S2. NCI fingerprint of selected molecules. Hinge region-related NCIs (i.e., E92 or C94 involved) are indicated with red boxes. More details are provided in Supplementary material 6.

*
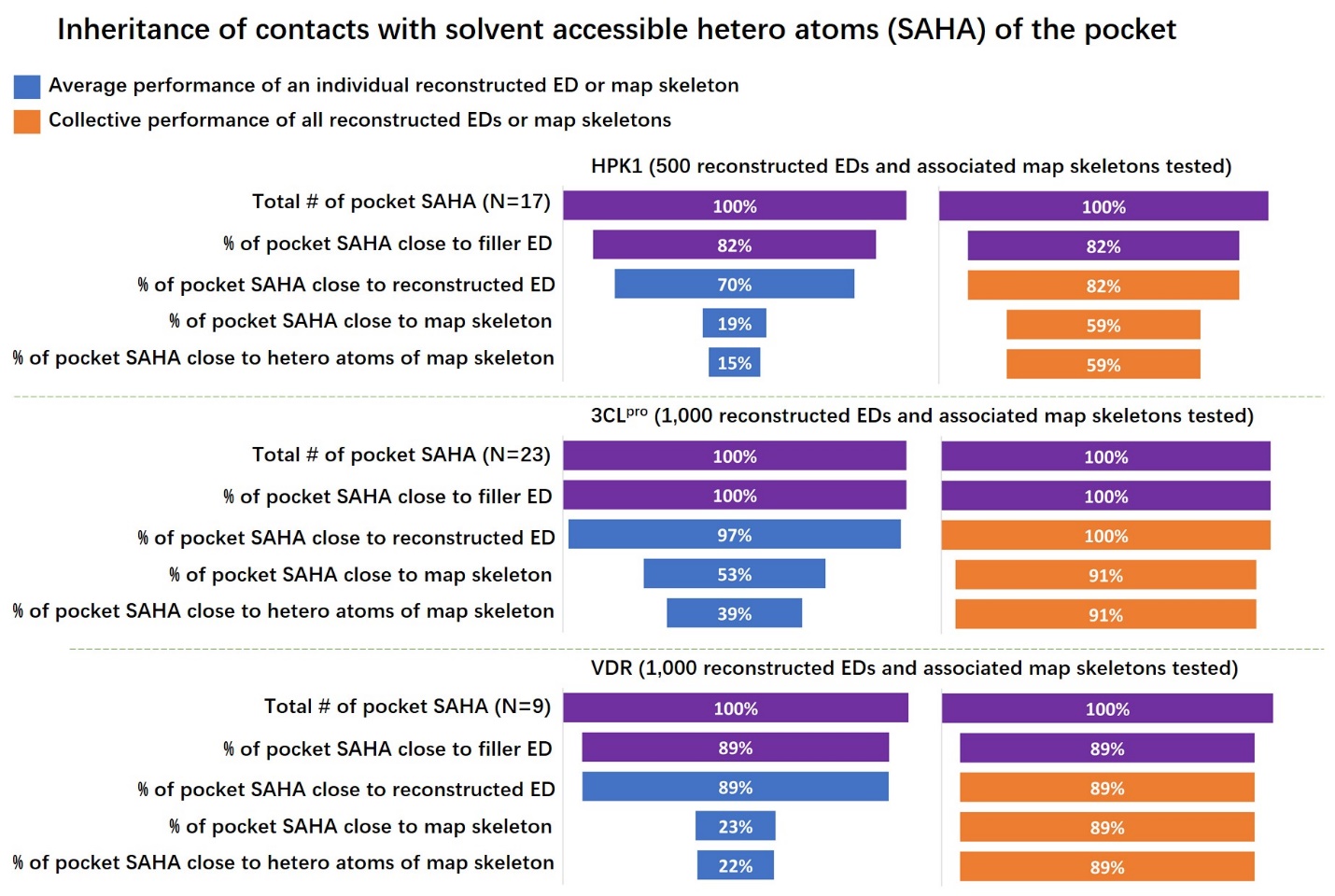
*

Figure S3. Inheritance of contacts with the solvent accessible hetero atoms (SAHA) of the pocket through different tasks. An atom of the pocket is defined as close to a target (e.g., a blob of ED or atoms in a map skeleton) if the distance between the atom and the target is less than 3.3 Å. Taking HPK1 as an example, its pocket possesses 17 SAHAs, and the filler ED can cover 14 SAHAs (82%). By analyzing 500 reconstructed EDs and the associated map skeletons, on average, one reconstructed ED covers 12 SAHAs (70%); one map skeleton covers 3.2 SAHAs (19%); and the hetero atoms in a map skeleton cover 2.6 SAHAs (15%). If we view the 500 reconstructed EDs as a group, then this group covers 14 SAHAs (82%). If we view the 500 map skeletons as a group, then this group covers 10 SAHAs (59%). If we view the hetero atoms of the 500 map skeletons as a group, then this group covers 10 SAHAs (59%).


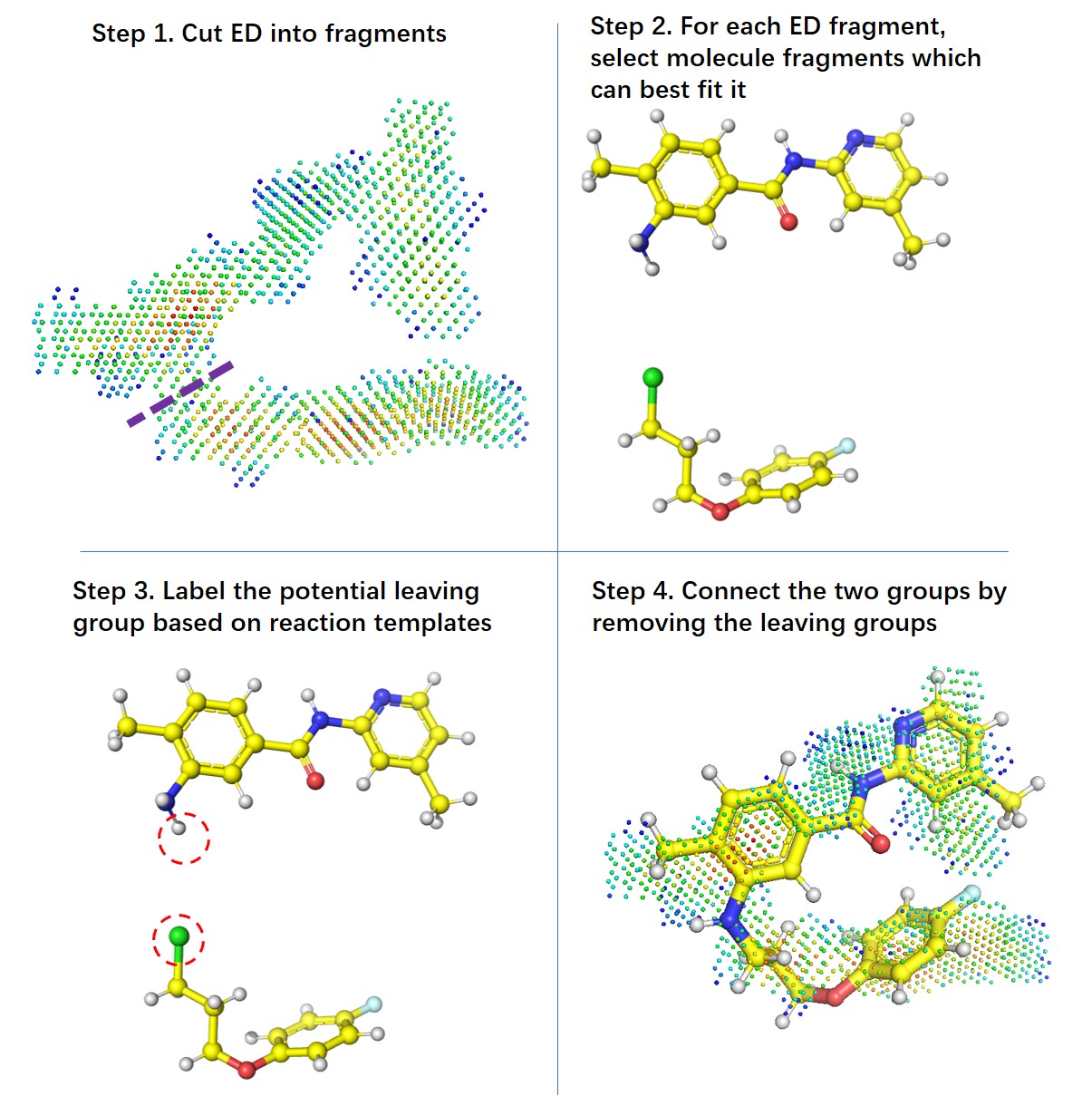


Figure S4. Illustration of molecular fragment assembly. One example is provided here, and more examples can be found in Table S5.

*
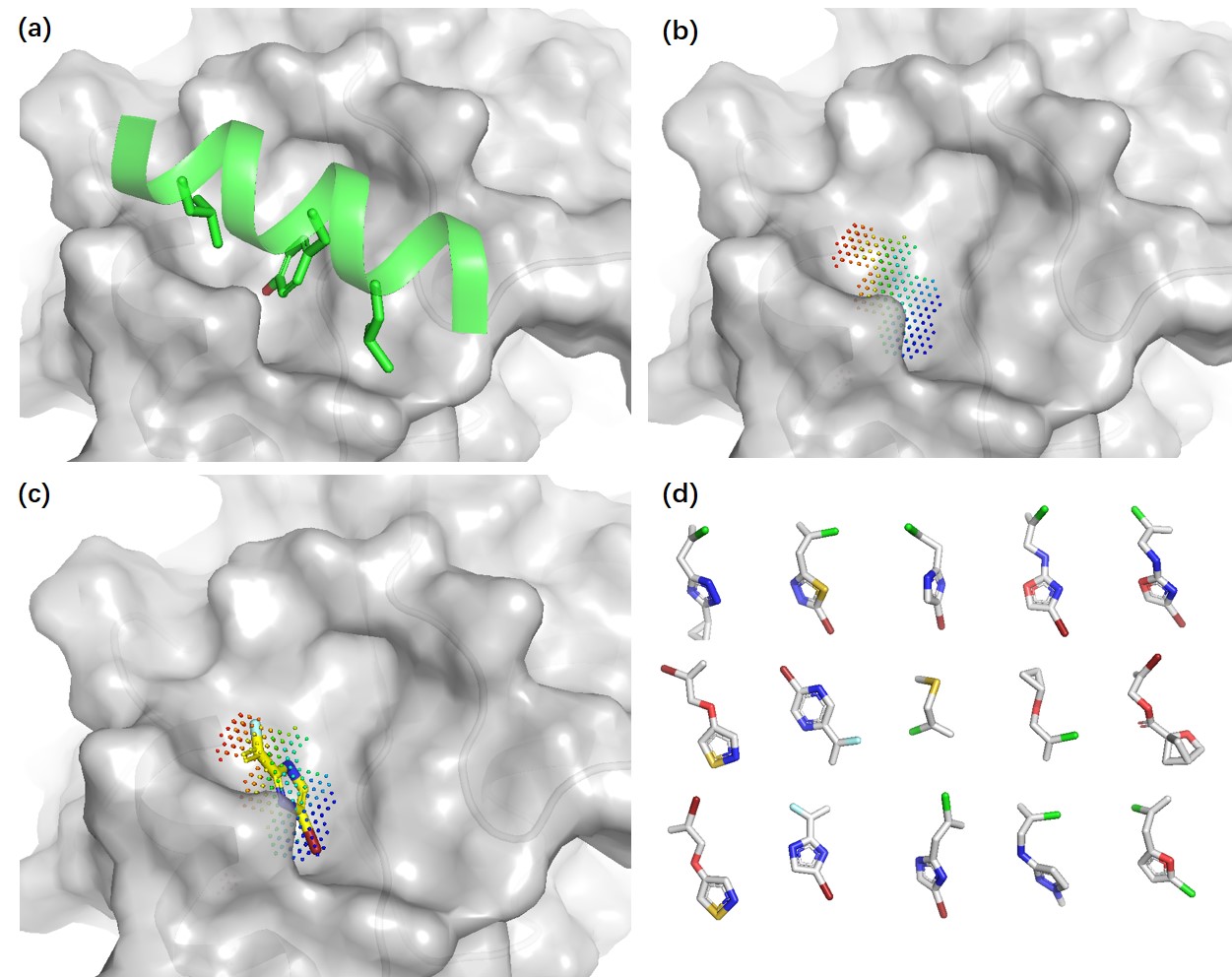
*

Figure S5. Recognizing small molecule binding regions in PPI interfaces. a) PPI of a protein interacting with a helix. The protein and helix are from PDB 1HH4 chain A (gray) and chain D (green), respectively. b) Generated ED using the PPI region from chain A (gray). c) Fragment-sized molecules generated based on the ED. d) More examples of generated molecules.
